# Subcutaneous Enoxaparin for Systemic Anticoagulation of COVID-19 Patients During Extracorporeal Life Support

**DOI:** 10.3389/fmed.2022.879425

**Published:** 2022-07-11

**Authors:** Marion Wiegele, Daniel Laxar, Eva Schaden, Andreas Baierl, Mathias Maleczek, Paul Knöbl, Martina Hermann, Alexander Hermann, Christian Zauner, Johannes Gratz

**Affiliations:** ^1^Department of Anesthesia, Intensive Care Medicine and Pain Medicine, Medical University of Vienna, Vienna, Austria; ^2^Ludwig Boltzmann Institute Digital Health and Patient Safety, Medical University of Vienna, Vienna, Austria; ^3^Department of Statistics and Operations Research University of Vienna, Vienna, Austria; ^4^Department of Medicine I, Medical University of Vienna, Vienna, Austria; ^5^Department of Medicine III, Medical University of Vienna, Vienna, Austria

**Keywords:** acute respiratory distress syndrome, bleeding, COVID-19, enoxaparin, extracorporeal membrane oxygenation, thrombosis

## Abstract

**Background:**

Extracorporeal membrane oxygenation, with an inherent requirement for anticoagulation to avoid circuit thrombosis, is a key element in the treatment of respiratory failure associated with COVID-19. Anticoagulation remains challenging, the standard of care being intravenous continuous administration of unfractionated heparin. Yet regimens vary. Some intensive care units in our center have successfully used enoxaparin subcutaneously in recent years and throughout the pandemic.

**Methods:**

We retrospectively analyzed adult COVID-19 patients with respiratory failure who had been systemically anticoagulated using either enoxaparin or unfractionated heparin. The choice of anticoagulant therapy was based on the standard of the intensive care unit. Defined thromboembolic and hemorrhagic events were analyzed as study endpoints.

**Results:**

Of 98 patients, 62 had received enoxaparin and 36 unfractionated heparin. All hazard ratios for the thromboembolic (3.43; 95% CI: 1.08–10.87; *p* = 0.04), hemorrhagic (2.58; 95% CI: 1.03–6.48; *p* = 0.04), and composite (2.86; 95% CI: 1.41–5.92; *p* = 0.007) endpoints favored enoxaparin, whose efficient administration was verified by peak levels of anti-factor Xa (median: 0.45 IU ml^−1^; IQR: 0.38; 0.56). Activated partial thromboplastin time as well as thrombin time differed significantly (both p<0.001) between groups mirroring the effect of unfractionated heparin.

**Conclusions:**

This study demonstrates the successful use of subcutaneous enoxaparin for systemic anticoagulation in patients with COVID-19 during extracorporeal membrane oxygenation. Our findings are to be confirmed by future prospective, randomized, controlled trials.

## Introduction

Life support by extracorporeal membrane oxygenation plays a major role in treating severe cases of acute respiratory distress syndrome (ARDS) associated with COVID-19 (1–4). It is generally accepted that anticoagulation of the extracorporeal circuit is mandatory to prevent systemic clotting and thromboembolic complications (5–7). At the same time, this requirement increases the risk of bleeding complications, which sometimes may be fatal (8, 9). To strike a correct balance between these potential complications, adequate dosing and efficient monitoring of the anticoagulant drug needs to be ensured (5, 6, 10).

There is ongoing discussion as to which anticoagulant medication has the best safety profile (11). Current guidelines recommend the use of unfractionated heparin for anticoagulation during extracorporeal membrane oxygenation (ECMO) (12). Standardized protocols using low-molecular-weight heparin have yet to find their way into routine clinical practice, even though promising data have been presented for enoxaparin and nadroparin (13–15). There are also reports on major advantages of low-molecular-weight over unfractionated heparin like reduced bleeding complications or heparin-induced thrombocytopenia (16, 17).

A number of intensive care units (ICUs) in our tertiary care center have come to use enoxaparin routinely for anticoagulation during ECMO therapy. The effectiveness of this approach was recently demonstrated in lung transplant patients (13) and we hypothesized, that this might also hold true for patients with COVID-19 associated ARDS. Throughout the COVID-19 pandemic, some of our ICUs continued to administer enoxaparin whereas others have used unfractionated heparin. This situation has enabled us to compare major (i) thromboembolic and (ii) bleeding complications in patients with severe COVID-19 who had received either enoxaparin subcutaneously or unfractionated heparin intravenously for anticoagulation during extracorporeal membrane oxygenation.

## Materials and Methods

### Study Overview

This study was an investigator-initiated, retrospective, observational cohort study. Study design as well as data handling and reporting followed the STROBE guidelines to obtain a maximum level of research quality (18). Patient data were collected from six ICUs associated with three departments (Anesthesia, Intensive Care Medicine and Pain Medicine; Medicine I; Medicine III) of our tertiary care center (University Hospital Vienna; Medical University of Vienna).

Approval was obtained from the institutional ethics committee (Medical University of Vienna; amendment to ID: 2024/2020 approved in 07/2021) and the study performed as required by applicable laws and regulations and the Helsinki Declaration. The need for informed consent was waived since this was an observational study and pseudonymized data were used for analysis. Adult (≥18 years) COVID-19 patients with acute respiratory failure were eligible who had been admitted to one of the six ICUs for extracorporeal membrane oxygenation between 1 March 2020, and 20 May 2021. Parameters of mechanical ventilation in some of the patients included in this study have recently been published (19).

**Figure 1:**
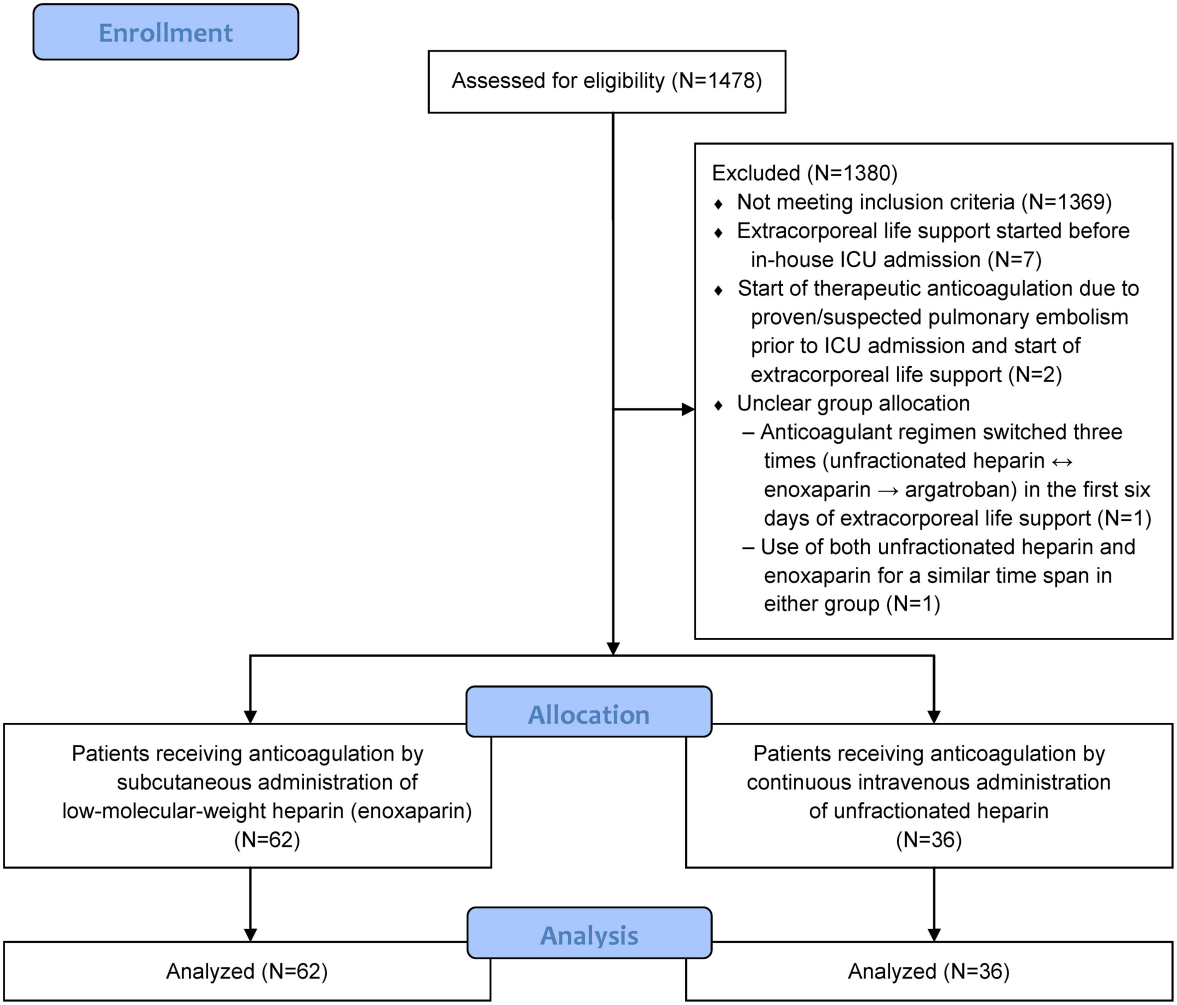
Enrollment and analysis.

We excluded patients whose extracorporeal circuit was anticoagulated by substances other than enoxaparin or unfractionated heparin, or in whom extracorporeal membrane oxygenation had been started before in-house ICU admission. Figure 1 gives a more detailed overview of how the patients were enrolled.

Available bed capacities had dictated to which one of the six ICUs dedicated to COVID-19 each patient was referred. The choice of anticoagulant therapy was based on the standard of the respective ICU. Key elements of COVID-19 therapy may be regarded as comparable, since the various departments involved followed international guidelines and also consented on Austrian recommendations as a common standard of care (20–22). ECMO therapy was provided as a last resort option after conventional treatment strategies (e.g., prone positioning, neuromuscular blockade) had failed or in the case of life-threatening hypoxia to avoid cardiopulmonary resuscitation (19). Criteria for patient eligibility, cannulation strategy and management during ECMO therapy have been summarized in a consensus statement of the Medical University of Vienna at the beginning of the pandemic (23). Intensive care units involved in the treatment of COVID-19 patients adhered to these recommendations. ECMO therapy was performed using the Cardiohelp® (Maquet Cardiopulmonary GmbH, Rastatt, Germany) or Novalung® (Xenios AG, Fresenius Medical Care, Heilbronn, Germany) device. A conventional transfusion trigger (hemoglobin level 7–9 g dl^−1^) was used to guide transfusion of packed red blood cells.

### Study Procedures

From the IntelliSpace Critical Care and Anesthesia (ICCA; Philips Healthcare, Amsterdam, Netherlands) patient data management system, we extracted pertinent details of patient demographics, extracorporeal membrane oxygenation, anticoagulation, and laboratory examinations. The latter included parameters of conventional coagulation assays, anti-factor Xa levels (peak and trough levels 3–5 and 12–14 h after administration of enoxaparin), and blood cell counts. Data on administered blood products (packed red blood cells, platelet concentrates, fresh frozen plasma) and procoagulant medications (antifibrinolytics, fibrinogen concentrates, prothrombin complex concentrate) were also retrieved. Because all patients had been sedated, a modified SOFA (sepsis-related organ failure assessment) score was used which excluded the Glasgow Coma Scale.

Two investigators (DL, MM) extracted information from the patient data management system by Structured Query Language. For each day in the ICU (defined as 4:00 to 3:59 a.m. to harmonize unit-specific workflows), mean values and standard deviations were calculated for continuous measurements and total values for drug doses. The extracorporeal circuits were coated with heparin and all patients received a bolus of 50–100 IU of unfractionated heparin per kilogram of body weight during cannulation (11). While this bolus was applied regardless of the subsequent anticoagulant regimen, all data analysis in the unfractionated heparin group is based on its continuous intravenous administration. Subcutaneous enoxaparin administration was initiated at 4000 IU twice daily (aiming for anti-factor Xa peak levels of 0.3–0.5 IU ml^−1^) and unfractionated heparin infusion adjusted based on activated partial thromboplastin time or anti-factor Xa levels, determined twice or three times daily and aiming for 50–60 s and / or 0.2–0.3 IU ml^−1^, respectively.

The same two investigators also performed automated screening both of the medical histories (for events predating the in-house ICU admissions) and of the daily clinical notes for keywords indicative of relevant thromboembolic or bleeding complications.

Details of the search strategy are given in the Supplementary Material (Additional File 1). In addition, a third investigator (MH) manually screened the entire ICCA documentation including the daily clinical notes and the ICU discharge reports, for any detailed relevant information.

### Endpoints and Observation Spans

Both patient groups (enoxaparin or unfractionated heparin) were assessed for occurrences of a primary and secondary endpoint during extracorporeal membrane oxygenation. As primary endpoint, clinically relevant thromboembolic events in the form of deep vein thrombosis, pulmonary embolism (excluding incidental findings of subsegmental pulmonary embolism) (24), heparin-induced thrombocytopenia, or pump / oxygenator / circuit exchanges were analyzed. The indications for pump / oxygenator / circuit exchanges were the same for all ICU and comprised the following: extracorporeal membrane circulation stop due to acute occlusion; visible clots within the system; and / or a significant drop in platelet count plus fibrinogen levels as a sign of active consumption. Major bleeding complications, which by definition of the International Society on Thrombosis and Haemostasis (ISTH) (25) include bleeding that requires surgery or transfusion of more than two units of packed red blood cells within 24 h but also any bleeding events into critical organs (i.e., brain) served as the secondary endpoint. This was supplemented by a composite endpoint of thromboembolic plus bleeding complications, as well as by tracking of relevant laboratory parameters (anti-factor Xa levels, activated partial thromboplastin time, thrombin time).

Observation spans were defined as time from cannulation to either (i) cessation of extracorporeal membrane oxygenation, (ii) occurrence of a defined endpoint event, (iii) switching to a different anticoagulant drug, or (iv) surgery requiring transfusion of > two units of packed red blood cells within 24 h. To avoid bias from temporary changes in anticoagulation regimens, only the first treatment cycle was included whenever extracorporeal membrane oxygenation, due to individual clinical developments, had been interrupted and resumed > 12 h later. As recommended by current guidelines (26), no routine screening for deep vein thrombosis had been performed. The study includes data recorded until 28 June 2021.

### Statistical Analysis

Whether both anticoagulant regimens made a difference in terms of thromboembolic events and bleeding complications was visualized by Kaplan-Meier curves and analyzed by Cox proportional hazards models. Observations with cessation of extracorporeal membrane oxygenation for reasons other than an endpoint event (i.e., death, improved clinical condition, or successful lung transplantation) were classified as right-censored. Cessation of ECMO therapy can be assumed unrelated to the occurrence of an event since there is no plausible link between the patient's health condition and the occurrence of an event. Proportional hazard assumptions were assessed visually and tested by diagnostics based on weighted residuals.

Differences in continuous demographic variables, baseline values, and intra-treatment laboratory assessments between both groups were descriptively expressed as median values along with first/third quartiles and assessed by Mann Whitney *U*-tests. Median values and interquartile ranges of the laboratory values obtained during extracorporeal oxygenation were derived from the medians of each patient's daily values. Dichotomous variables were analyzed by calculating per-group percentages and χ^2^-tests. Dichotomous variables with multiple records per patient were first aggregated on patient level by deriving proportions. Subsequently, mean proportions per group were compared by Mann Whitney *U*-tests.

*P*-values were considered significant if < 0.05 and, for the secondary (i.e., the hemorrhagic) endpoint events, were adjusted for multiplicity by Holm's procedure. Environments that were used for statistical processing of the data included Python 3.8 (27), Pandas 1.1.3 (28), and R 4.0.2 (29).

## Results

Ninety-eight patients could be evaluated, 62 of whom had received enoxaparin and 36 unfractionated heparin. A flow chart of the study is presented in Figure 1. As shown in Table 1, the baseline patient demographics were comparable in both groups, with the exception of sepsis-related organ failure assessments (*p* = 0.04). Baseline laboratory parameters (conventional coagulation parameters, anti-factor Xa levels, platelet counts, hemoglobin levels) did not differ significantly, except for thrombin time (*p* = 0.02) that was documented for only 46 patients at values within reference ranges. Additional File 2 in the Supplementary Material presents further details with regard to general treatment, transfusion triggers, transfusion requirements and administration of coagulation factor concentrates.

**Table 1:** Baseline data and baseline laboratory findings.

	**Low-molecular-weight (enoxaparin) vs. unfractionated heparin**			
**Characteristic**		**Enoxaparin (*N* = 62)**	**Unfractionated (*N* = 36)**	***P-*Value**
**Patient demographics**				
Age, median (IQR) — yr		57 (53–62.8)	57 (50.8–61)	0.35
Male sex — no. (%)		48 (77)	21 (58)	0.08
Body mass index, median (IQR)		29.2 (26.3–36.3)	30.9 (26.7–33.3)	0.82
SOFA score, median (IQR)		7.0 (6.0–8.0)	8.0 (7.0–9.0)	0.04
Length of ICU stay, median (IQR) — d^a^		31 (19–46)	35 (23–59)	0.18
ICU mortality — no (%)^a^		28 (45.2)	11 (30.5)	0.30
**Baseline laboratory values**, median (IQR)	N			
Anti-factor Xa level — IU ml^−1^ ^b^	51	0.29 (0.23–0.35)	0.36 (0.25–0.57)	0.07
Activated partial thromboplastin time — s	97	40.2 (34.8–45.6)	41.4 (38.6–49.4)	0.13
Thrombin time — s	46	17 (15.7–18.3)	19.6 (17.4–24.8)	0.02
Antithrombin — %	97	87.0 (76.0–98.0)	85.0 (67.8–95.8)	0.20
Prothrombin time (Owren method) — %	97	78.0 (66.3–88.0)	73.0 (62.8–89.6)	0.44
Fibrinogen (Clauss method) — mg dl^−1^	98	708 (560–850)	647 (518–734)	0.18
D-dimer — μl ml^−1^	66	3.35 (2.51–6.79)	3.97 (2.07–6.40)	0.69
Platelet count — G l^−1^	97	239 (169–313)	218 (170–285)	0.36
Hemoglobin — g dl^−1^	98	9.7 (9.1–10.4)	9.4 (8.6–9.9)	0.13
Hematocrit — %	98	29.9 (28.1–32.3)	29.0 (26.5–30.6)	0.12

*SOFA, sepsis-related organ failure assessment scores obtained within 24 h of ICU admission*.

a *Five patients were still admitted to the ICU at the end of the observation period, and three patients were lost to follow-up (transferred to another hospital);*

b *baseline value irrespective of time since drug administration*.

*Reference ranges and SI conversion factors: anti-factor Xa (RR: < 0.1 IU ml^−1^) | activated partial thromboplastin time (RR: 27–41 s; SI: 1) | thrombin time (RR: < 21 s; SI: 1) | antithrombin (RR: 80–120%; SI: 0.01) | prothrombin time (RR: 70–125%; SI: 0.01) | fibrinogen (RR: 200–400 mg dl^−1^; SI: 0.01) | D-dimer (RR: < 0.5 μl ml^−1^; SI: 5.476) | platelets (RR: 150–350 G l^−1^) | hemoglobin (RR: 12–18 g dl^−1^; SI: 10) | hematocrit (RR: 35–52 SI: 0.01)*.

Except for one patient in the LMWH group, all patients underwent prone positioning during the observational period. Median paO2/FiO2 ratio prior to the start of ECMO therapy was 72.2 mmHg (IQR: 60.5–96.6) in the LMWH group and 74.2 mmHg (IQR: 66.3–155.7) in the UFH group (*p* = 0.04). Extracorporeal membrane oxygenation was provided over 1,741 patient days (until the occurrence of an event). Venovenous cannulation accounted for the majority of patients in both groups, and venoarterial or venovenoarterial, as used in situations of combined cardiac and respiratory failure, for the remainder (Table 2). The group-specific median durations of these therapies were comparable (*p* = 0.09). Endpoint events are listed in Table 2. A total of 35 events would per se have terminated the observation span, but given two coinciding events in two cases, only 33 patients were affected: one diagnosed with intracerebral bleeding and major bleeding requiring > two units of packed red blood cells within 24 h, and one with heparin-induced thrombocytopenia in addition to requiring an oxygenator exchange, each on the same day in the ICU.

**Table 2:** Endpoint events and laboratory parameters.

		**Low-molecular-weight (enoxaparin) vs. unfractionated heparin**		
**Characteristic**		**Enoxaparin (*N* = 62)**	**Unfractionated (*N* = 36)**	***P-*Value**
**Extracorporeal membrane oxygenation**				
Duration, median (IQR) — d		17 (10–28)	23 (13–45)	0.09
Days on venovenous mode, mean — %^a^		98.7	93.9	0.55
Mortality during oxygenation — n (%)		20 (32.2)	10 (38.5)	0.81
Blood flow, median (IQR) — l min^−1*b*^		3.22 (2.80–3.80)	3.82 (3.48–4.16)	0.002
**Anticoagulant therapy**, median (IQR)				
Daily cumulative dosage — IU d^−1^		8,000 (8,000–10,000)	21,925 (15,213–26,813)	n/a
**Patients meeting endpoint events** — ***n*** **(%)**		13 (21.0)	20 (55.6)	n/a
Pulmonary embolism		2 (3.2)	0 (0)	n/a
Deep vein thrombosis		0 (0)	0 (0)	n/a
Heparin induced thrombocytopenia		0 (0)	1 (2.8)	n/a
Exchange of oxygenator		3 (4.8)	8 (22.2)	n/a
Major bleeding		7 (11.3)	8 (22.2)	n/a
Bleeding into critical organ^c^		1 (1.6)	5 (13.9)	n/a
**Laboratory parameters**, median (IQR) ^b^	N			
Anti-factor Xa level — IU ml^−1^ ^d^	36	n/a	0.27 (0.25–0.28)	n/a
Anti-factor Xa level (peak) — IU ml^−1^	56	0.45 (0.38–0.56)	n/a	n/a
Anti-factor Xa level (trough) — IU ml^−1^	31	0.39 (0.30–0.51)	n/a	n/a
Activated partial thromboplastin time — s	98	43.4 (38.1–48.9)	55.8 (44.8–60.2)	<0.001
Thrombin time — s	64	18.5 (17–22.2)	35.0 (26.8–43.3)	<0.001
Antithrombin — %	98	99 (90–119)	78 (70–88)	<0.001
Prothrombin time (Owren method) — %	98	81 (73–92)	79 (69–91)	0.63
Fibrinogen (Clauss method) — mg dl^−1^	98	508 (381–596)	554 (457–618)	0.16
D-dimer — μl ml^−1^	82	7.0 (2.99–15.1)	5.46 (3.52–8.37)	0.17
Platelet count – G l^−1^	98	159 (129–207)	163 (105–223)	0.84
Hemoglobin — g dl^−1^	98	9.5 (8.9–9.9)	9.2 (8.9–9.5)	0.26
Hematocrit — %	98	29.4 (27.7–30.5)	28.5 (27.8–28.5)	0.27

a *Derived from daily means;*

b *derived from daily medians;*

c *intracranial in all cases;*

d *irrespective of time since drug administration. References ranges and SI conversion factors: anti-factor Xa (RR: < 0.1 IU ml^−1^) | activated partial thromboplastin time (RR: 27–41 s; SI: 1) | thrombin time (RR: < 21 s; SI: 1) | antithrombin (RR: 80–120%; SI: 0.01) | prothrombin time (RR: 70–125%; SI: 0.01) | fibrinogen (RR: 200–400 mg dl^−1^; SI: 0.01) | D-dimer (RR: < 0.5 μl ml^−1^; SI: 5.476) | platelets (RR: 150–350 G l^−1^) | hemoglobin (RR: 12–18 g dl^−1^; SI: 10) | hematocrit (RR: 35–52 SI: 0.01)*.

**Figure 2:**
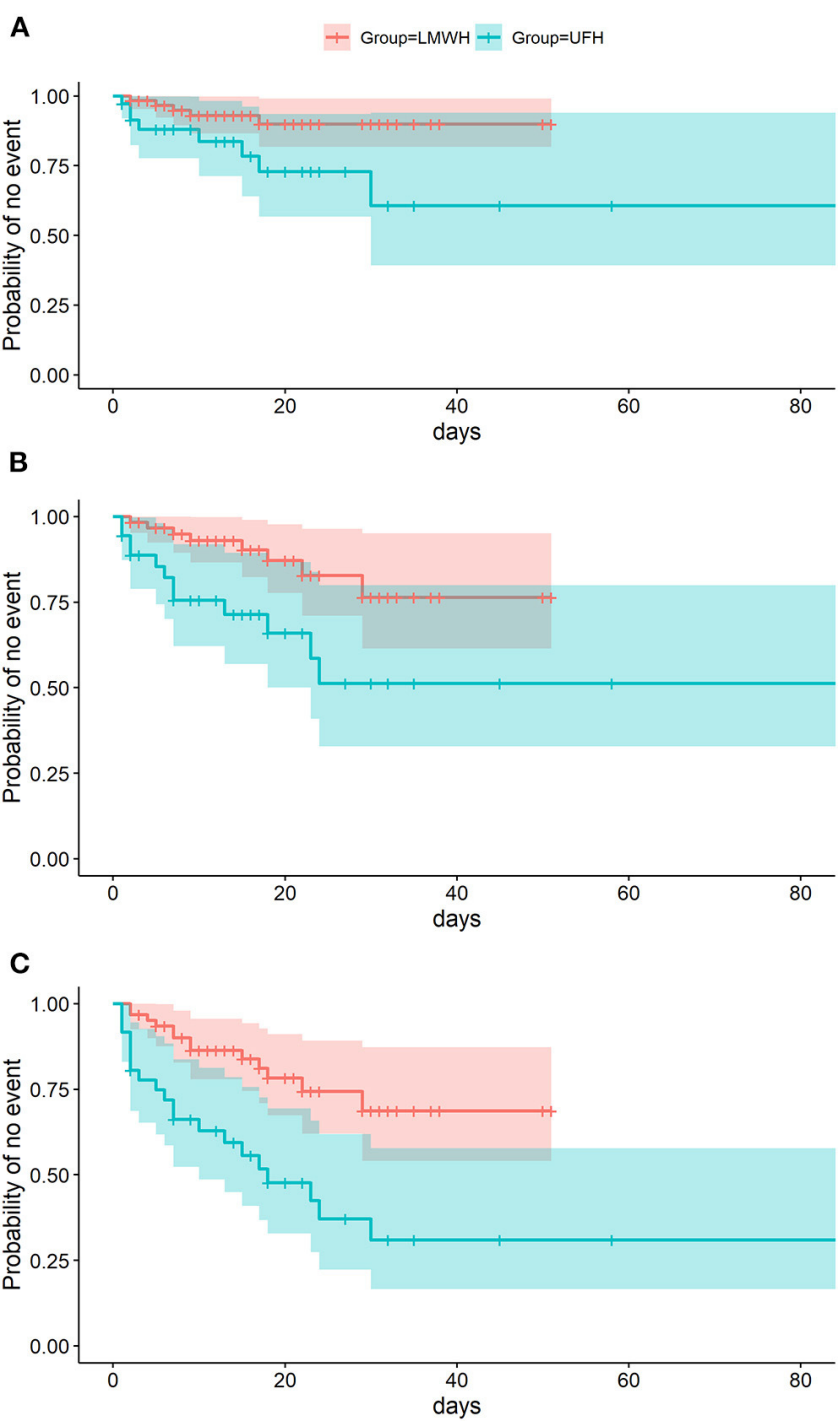
Event-free days during extracorporeal membrane oxygenation. Events that terminated the observation span included **(A)** thromboembolic endpoint events, **(B)** hemorrhagic endpoint events, and **(C)** a composite endpoint made up of both.

All endpoint events, with the exception of pulmonary embolism, were found to have occurred less frequently in the enoxaparin group (Table 2 and Figure 2). No clinically relevant cases of deep vein thrombosis were noted. Figure 2 illustrates the timelines of event-free days per endpoint per group. Adjustment in calculating the hazard ratios was required by the baseline findings, listed in Table 1, of a significant difference in sepsis-related organ failure assessments (*p* = 0.04) and a certain difference in gender distribution (*p* = 0.07).

**Figure 3:**
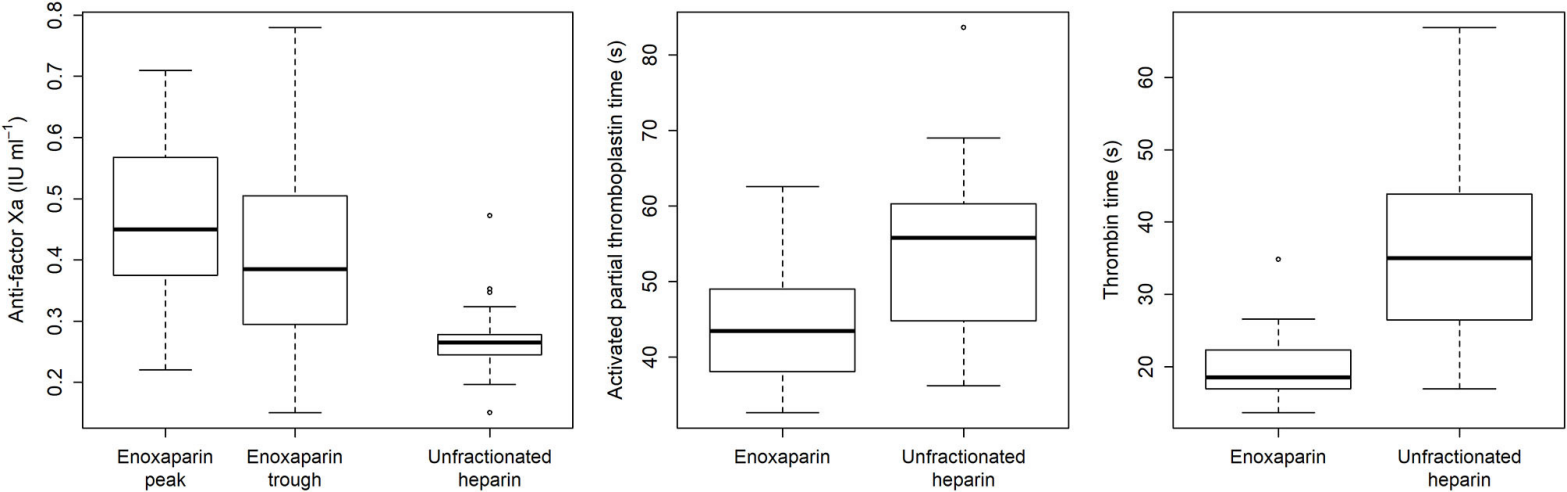
Laboratory parameters used to monitor the anticoagulant medications during extracorporeal membrane oxygenation.

All adjusted hazard ratios favored enoxaparin over unfractionated heparin: 3.43 for the thromboembolic primary endpoint (95% CI: 1.08–10.87; *p* = 0.04); 2.58 for the hemorrhagic secondary endpoint (95% CI: 1.03–6.48; *p* = 0.04); and 2.86 for the composite endpoint of both event types (95% CI: 1.41–5.92; *p* = 0.007). As shown in Table 2 and Figure 3, efficient dosing of enoxaparin was reflected by peak levels of anti-factor Xa (median: 0.45 IU ml^−1^;IQR: 0.38; 0.56). Activated partial thromboplastin time and thrombin time differed significantly (*p* < 0.001) between the groups mirroring the effect of unfractionated heparin.

## Discussion

This is the first study to report on the successful use of the low-molecular-weight heparin enoxaparin for anticoagulation in COVID-19 patients with acute respiratory distress syndrome during extracorporeal membrane oxygenation. Compared to unfractionated heparin, we found that enoxaparin was associated with superior results in terms of both clinically relevant thromboembolic events and major bleeding complications.

Current guidelines recommend continuous intravenous administration of unfractionated heparin for systemic anticoagulation to prevent intra- and extracorporeal clotting during extracorporeal membrane oxygenation (12). Even with this recommendation implemented, 19 to 50% rates of thromboembolic events have recently been reported in scenarios of respiratory distress syndrome or lung transplantation, compounded by 19 to 66% rates of transfusion due to hemorrhagic side effects of high-dose anticoagulation (10, 13, 30, 31). The present study follows suit in reporting a 25% rate of thromboembolic and a 34% rate of bleeding complications despite the use of unfractionated heparin as recommended.

Given these high complication rates associated with unfractionated heparin, there has been increasing interest lately in low-molecular-weight heparin, with reported rates of 6.5 or 20% for thromboembolic and 12.5 or 30% of bleeding complications (13, 14). Consistent with these figures, the use of enoxaparin in the present study involved 8% thromboembolic and 12.9% bleeding complications. Krueger et al. (14) were the first to report on enoxaparin, administered subcutaneously in standard prophylactic doses, for venovenous extracorporeal oxygenation in acute respiratory distress syndrome, with thrombosis of the centrifugal pump occurring in 5% of patients during the first week. This rate is very similar to the 4.8% rate of oxygenator exchanges in our enoxaparin group, compared to 22.3% in the unfractionated-heparin group (see Table 2).

Only two direct comparisons are currently available on the safety and efficacy of low-molecular-weight vs. unfractionated heparin during extracorporeal oxygenation. While Piwowarczyk et al. (15) noted similar rates of thromboembolic events and bleeding complications for nadroparin and unfractionated heparin, these findings might not be directly relatable to ours, since the comparability of enoxaparin and nadroparin has been questioned (32). Our own study group observed, in perioperative settings of lung transplantation, bleeding events with enoxaparin in 12.5% and with unfractionated heparin in 22.7% of patients (*p* = 0.31) (13). Our finding herein reported of similar rates (11.3 or 22.2%, respectively; see Table 2) in spite of median daily cumulative dosages of enoxaparin nearly twice as high (4,800 vs 8,000 IU) might point to an inherently increased risk of bleeding in our previous cohort of perioperative transplant patients.

Increasing the risk of heparin-induced thrombocytopenia is a major drawback of unfractionated compared to low-molecular-weight heparin. In the present study, one such diagnosis was made in the unfractionated-heparin group, which, given a 0.5% overall incidence during critical illness (33, 34), corresponds to a notable 2.8% rate in 36 patients. An increased prevalence of heparin-induced thrombocytopenia during extracorporeal membrane oxygenation has been reported previously (35, 36). Also, Daviet et al. recently reported an almost 10-fold increase in positivity for heparin-induced thrombocytopenia, up to a prevalence of 8%, with unfractionated heparin in COVID-19 patients, due to reasons possibly consisting in higher drug concentrations needed for therapeutic anticoagulation and COVID-19-related exacerbation of immune reactions (37).

The Extracorporeal Life Support Organization recommends a multimodal approach to anticoagulation monitoring by adjusting unfractionated heparin based on appropriate standard curves for activated partial thromboplastin time, activated clotting time, thrombin time, or anti-factor Xa levels (12). The anticoagulant regimens herein reported had been guided accordingly, with a 0.45 IU ml^−1^ median peak level of anti-factor Xa (IQR: 0.38; 0.56) proving the activity of enoxaparin, and the one of unfractionated heparin mirrored by significant differences from enoxaparin for the activated partial thromboplastin time (*p* < 0.001).

A few limitations of our study should be noted. First of all, retrospective findings will always carry some risk of bias, although we processed all existing data, missing data, and potentially incomplete records with meticulous care. As a case in point, the threshold for oxygenator exchanges was progressively lowered throughout the pandemic while our automated system of patient documentation did not disclose in each specific instance whether clotting of the oxygenator had actually occurred or been imminent. Second, most patients had been referred from hospitals using various anticoagulant regimens. Hence, to avoid potential confounders, we only included patients with complete documentation of anticoagulant medication after in-house cannulation. Third, no routine screening for thromboembolic events had taken place, although this should not formally be regarded as a limitation given that current guidelines recommend against such screening for venous thromboembolism in critically ill patients (26). Still, this lack of screening might account for the small total number of these complications in our study despite a median duration of extracorporeal membrane oxygenation of more than 2 weeks. Lastly, it is important to note that complication rates in terms of thromboembolism and bleeding might vary depending on the ECMO cannulation mode.

In summary, subcutaneously administered enoxaparin is a feasible anticoagulation strategy for extracorporeal membrane oxygenation in COVID-19 patients. Both the thromboembolic primary and the hemorrhagic secondary endpoint of our study yielded results of this approach superior to unfractionated heparin. Jumping to definite conclusions based on this retrospective analysis would be ill-advised, but the current data at the very least support the effective use of enoxaparin in this vulnerable patient cohort and highlight the urgent need for prospective, randomized trials.

## Data Availability Statement

The original contributions presented in the study are included in the article/Supplementary Material, further inquiries can be directed to the corresponding author.

## Ethics Statement

The studies involving human participants were reviewed and approved by Medical University of Vienna; ID: 2024/2020. Written informed consent for participation was not required for this study in accordance with the national legislation and the institutional requirements.

## Author Contributions

MW, ES, and JG conceived and designed the study, interpreted data, and drafted the manuscript. DL and MM acquired the data. AB performed statistical analysis and interpreted data. PK interpreted data. Critical revisions of the manuscript, in addition to all of the aforementioned authors, were contributed by AH, MH, and CZ. All authors commented on successive versions and have read and approved the final manuscript.

## Funding

This work was supported by departmental funds of the Department of Anesthesia, Critical Care and Pain Medicine Division of General Anesthesia and Intensive Care Medicine, Medical University of Vienna and the Ludwig Boltzmann Institute Digital Health and Patient Safety, Medical University of Vienna, Vienna, Austria.

## Conflict of Interest

MW received honoraria, research funding and travel reimbursement from Boehringer Ingelheim, CSL Behring and Mitsubishi Tanabe Pharma. JG received honoraria, research funding, and travel reimbursement from Alexion, Boehringer Ingelheim, CSL Behring, Johnson & Johnson, Instrumentation Laboratory, Mitsubishi Tanabe Pharma, Octapharma, and Portola. The remaining authors declare that the research was conducted in the absence of any commercial or financial relationships that could be construed as a potential conflict of interest.

## Publisher's Note

All claims expressed in this article are solely those of the authors and do not necessarily represent those of their affiliated organizations, or those of the publisher, the editors and the reviewers. Any product that may be evaluated in this article, or claim that may be made by its manufacturer, is not guaranteed or endorsed by the publisher.
