# Supplementary material for: Subcutaneous Enoxaparin for Systemic Anticoagulation of COVID-19 Patients During Extracorporeal Life Support
Source: Front Med (Lausanne). 2022 Jul 11;9:879425. doi: 10.3389/fmed.2022.879425 (PMC9309531; doi:10.3389/fmed.2022.879425)
Supplement: Supplementary file 1 [file Data_Sheet_1.PDF]

Supplementary Material

“Subcutaneous Enoxaparin for Systemic Anticoagulation of Covid-19 Patients during Extracorporeal Life Support”

Additional file 1

1 Data Inclusion

Patients admitted to an ICU of the Department of Anesthesia and Intensive Care, Internal Medicine I or Internal Medicine III between 2020-03-01 and 2021-05-20 were included. Data recorded until 2021-06-28 was extracted from our electronic health records (EHR) via SQL.

2 Data Processing

The exported data was then filtered and processed for statistical analysis using Python 3.8 (primarily pandas 1.1.3.)

3 Data Screening

Clinical notes were filtered by keywords and then screened manually. The German keywords as well as their translations are listed below:

**German Keywords**	**English Translation**
Blut EXCEPT Blutfl	Blood EXCEPT bloodst (as in *blood*stain)
Hämorrh	haemorrh
Transfusion	Transfusion
Konserve	Blood product
Ischäm	Ischem
Minderdurchbl	Lack of perfusion
Livid	Livid
Thromb	Thromb
Pulmonalembolie	Pulmonary embolism

*[Space]*PE*[Space]*	*[Space]*PE*[Space]*
PAE	Pulomary artery embolism, PAE
Oxygenatortausch	Change of oxygenator
Oxy **EXCEPT** Oxygenierung	Oxy **EXCEPT** Oxygenation
Clot	Clot
Beinkanüle	Femoral cannula
Fogarty	Fogarty
Beinvene	Leg vein
TVT	Deep Venous Thrombosis, DVT
ICB	Intracranial Bleeding, ICB
Raumfor	start of the word describing a tumor

Matches were made case-insensitively. Keywords in the form “a **EXCEPT** b” did only match if the text did not match the keyword *b* but matches the keyword *a*.

Keywords are insensitive to characters before and after the start of the keyword. This means that the keyword “PE” would match “su**pe**r” as well as “the CT showed a massive **PE** on the left side”, which is why some keywords contains whitespace characters (denoted as *[Space]*) to only match single words.

All matches of at least one keyword were then screened manually for adverse events.

4 Adverse Events

Adverse events were imported from the screened matches. Adverse events such as surgical interventions were imported directly from the electronic health records (EHR).
