# Supplementary material for: Subcutaneous Enoxaparin for Systemic Anticoagulation of COVID-19 Patients During Extracorporeal Life Support
Source: Front Med (Lausanne). 2022 Jul 11;9:879425. doi: 10.3389/fmed.2022.879425 (PMC9309531; doi:10.3389/fmed.2022.879425)
Supplement: Supplementary file 2 [file Data_Sheet_2.pdf]

Supplementary Material

“Subcutaneous Enoxaparin for Systemic Anticoagulation of Covid-19 Patients during Extracorporeal Life Support”

Additional file 2

Additional information on general treatment and transfusion / substitution politics

		Low-molecular-weight (enoxaparin) versus unfractionated heparin		
**Characteristic**		**Enoxaparin (N=62)**	**Unfractionated (N=36)**	**P Value**
**General treatment**	**N**			
Fluid balance per day (ml)[a]	98	140.3 (-83.3−559.0)	482.7 (218.2−716.8)	0.048
Hemoglobin level leading to PRBC transfusion, median (IQR) — g dl^{-1}	94	8.4 (7.9−8.7)	7.9 (7.6−8.2)	0.012
**Transfusion requirements**				
Packed red blood cells — %[b]	98	95.2	97.2	1
Platelet concentrate — %[b]	98	4.8	11.1	0.45
Fresh frozen plasma — %[b]	98	0	5.6	0.26
**Coagulation factor concentrates**				
Prothrombin complex concentrates[c] — %[b]	98	8.1	0	0.20
Fibrinogen concentrate — %[b]	98	29.0	2.8	0.004
Antithrombin — %[b]	98	1.6	19.4	0.006

[a] daily fluid balance is routinely calculated from 6:00 to 6:00 a.m. by the automated patient data management system
[b] percentage of patients with at least one administration during the observational period
[c] either 4-factor PCC or Cofact® (for patients with heparin-induced thrombocytopenia [HIT])
